# Under-Balcony Acoustic Diagnosis Using FOA-Based Directional Metrics: Early–Late Entropy and Vertical-Energy Discrepancy at 125 Hz, 1 kHz, and 4 kHz

**DOI:** 10.3390/s26061871

**Published:** 2026-03-16

**Authors:** Po-Chun Ting, Yu-Cheng Liu

**Affiliations:** 1 Ph.D. Program of Mechanical and Aeronautical Engineering, Feng Chia University, Taichung City 407102, Taiwan; ericsson0925@hotmail.com; 2 Master Program of Electro-Acoustics, Feng Chia University, Taichung City 407102, Taiwan

**Keywords:** first-order ambisonics (FOA), room impulse response (RIR), active acoustic intensity, direction-of-arrival (DoA) estimation, directional spatial entropy, under-balcony acoustics, vertical early-reflection deficiency, frequency-dependent shadowing, spatial mapping, concert hall acoustics

## Abstract

Traditional concert-hall evaluations primarily rely on ISO 3382-1 scalar parameters (e.g., C_50_ and C_80_), which summarize temporal energy behavior but provide limited insight into the directional composition of early reflections, particularly in geometrically shadowed seating zones. This paper presents a first-order Ambisonics (FOA)-based 3D acoustic sensing framework to diagnose under-balcony directional imbalance, with emphasis on early vertical-reflection deficiency. Scene-based FOA impulse responses (WXYZ) were measured at 11 audience positions (P1–P11) in the National Concert Hall (Taipei) and analyzed using intensity-based direction-of-arrival (DoA) proxies, axis-resolved directional energy build-up, and a distributional descriptor based on directional spatial entropy. Results are presented at three representative frequencies (125 Hz, 1 kHz, and 4 kHz) and analyzed within full (0–200 ms), early (0–80 ms), and late (80–200 ms) windows. While the magnitude proxy $|p^{meas}(f)|$ exhibits strong seat-to-seat variability and does not support a uniform attenuation assumption under the balcony, direction-resolved metrics reveal a consistent under-balcony signature. Specifically, the early–late vertical energy discrepancy $\Delta R_{z}=R_{z}^{early}-R_{z}^{late}$ is persistently negative at under-balcony positions (P7–P11) across all three frequencies, indicating a selective reduction in early vertical contribution relative to the late field. Directional entropy analysis further shows predominantly negative $\Delta H_{n}=H_{n}^{early}-H_{n}^{late}$, with more negative values in the under-balcony group, consistent with stronger early directional constraint in shadowed seats. Spatial trend maps are provided via Gaussian RBF interpolation within the audience domain for visualization only. The proposed FOA-based diagnostic framework provides a practical and physically interpretable approach to identify direction-specific early-reflection deficits that remain masked in conventional scalar evaluations, supporting mechanism-oriented assessment and targeted intervention in geometrically constrained listening areas.

## 1. Introduction

Acoustic performance in concert halls is typically evaluated using scalar parameters derived from omnidirectional room impulse responses (RIRs). The standardized procedures and parameter definitions specified in ISO 3382-1 enable reproducible benchmarking across venues and measurement campaigns [1,2]. While these metrics capture the temporal aspects of energy decay and clarity, they do not explicitly describe the directional distribution of reflections, which is essential to spatial impression and listener perception in music performance spaces [3,4]. Both classical and contemporary studies have shown that early reflection directionality—particularly lateral components—plays a central role in perceived spaciousness and apparent source width [3,4,5].

A persistent seat-dependent issue in auditoria is acoustic degradation in under-balcony regions. Balcony overhangs alter reflection availability and reduce late energy in shadowed zones. The magnitude and characteristics of this degradation depend on local geometry, including the soffit and rear-wall configuration [6]. Consequently, under-balcony problems are often not limited to overall level reduction but involve directional deficits and altered early/late energy balance, which may not be adequately captured by conventional scalar metrics [1,2,3,6].

Recent spatial measurement and analysis methods provide practical tools to address these limitations. Scene-based microphone techniques, particularly first-order Ambisonics (FOA), represent the local sound field using four orthogonal components (W,X,Y,Z), supporting direction-of-arrival (DoA) estimation through intensity-based analysis and time–frequency directional descriptors [7,8,9,10]. Related parametric frameworks for spatial impulse response analysis, including Spatial Impulse Response Rendering (SIRR) and Directional Audio Coding (DirAC), operationalize DoA and diffuseness estimation within frequency bands [7,8,9,10]. In addition, the Spatial Decomposition Method (SDM) encodes spatial RIRs into reflection-like components suitable for analysis and rendering [11,12,13]. While these methods enable direction-resolved characterization at discrete receiver positions, meaningful seat-to-seat comparison still benefits from compact distributional metrics that quantify how concentrated or spread the directional energy is.

Information-theoretic measures provide a principled means to quantify directional complexity. Shannon entropy characterizes uncertainty in a discrete distribution and can be applied to the angular energy distribution of DoA samples. In this context, entropy indicates whether energy is broadly distributed (diffuse-like) or concentrated in a few directions (anisotropic dominance) [14,15,16]. Such an entropy-based descriptor complements axis-wise energy summaries and intensity/energy diffuseness indicators by explicitly capturing distributional structure.

Another limitation in large-venue field studies is the sparsity of spatial sampling. Practical measurement campaigns often rely on a limited number of receiver positions, and geometry-free interpolation does not enforce acoustic physics. Physics-Informed Neural Networks (PINNs) embed governing equations (e.g., wave/Helmholtz equations) into the learning objective and have been demonstrated as effective tools for field reconstruction from limited observations [17,18,19]. These developments suggest a viable route toward continuous field mapping that remains consistent with propagation physics. 

### 1.1. Research Motivation and Gap

Under-balcony seats remain a representative case of geometry-driven, seat-dependent degradation in concert halls [6]. Standard ISO 3382-1 parameters support global benchmarking but are not designed to diagnose directional deficits, such as reduced vertical early-reflection energy or directionally concentrated reflection patterns under overhangs [1,2,3,6]. Direction-resolved FOA/parametric methods (e.g., intensity-based DoA estimation, SIRR/DirAC, SDM) provide reflection-structure cues and directional features [7,8,9,10,11,12,13]. However, two practical gaps remain: (i) The absence of a compact metric capable of a summarizing directional-distribution structure across seats and time–frequency windows. (ii) The lack of a physics-consistent framework for interpreting sparse measurements spatially, rather than only point-wise, under limited receiver coverage [17,18,19].

### 1.2. Objective and Contribution

To bridge these gaps, this study aims to diagnose under-balcony directional deficits using sparse FOA measurements and to provide interpretable indicators and physics-consistent spatial mappings. The main contributions of this work are as follows:

1. FOA directional diagnostics: active-intensity-based DoA analysis with time-resolved hedgehog visualization and axis-resolved directional energy build-up over 0–200 ms for cross-seat comparison [7,8].
2. Directional spatial entropy: a normalized Shannon-entropy metric computed from binned angular energy distributions to quantify concentration versus spread of directional energy in selected time–frequency windows [14,15,16].
3. Physics-regularized reconstruction: a frequency-domain PINN constrained by the Helmholtz equation to reconstruct continuous pressure-field maps from sparse receiver positions and support spatial interpretation of under-balcony shadowing [17,18,19].

## 2. Methodology

### 2.1. Receiver Layout and Spatial Reference

**Figure 1:**
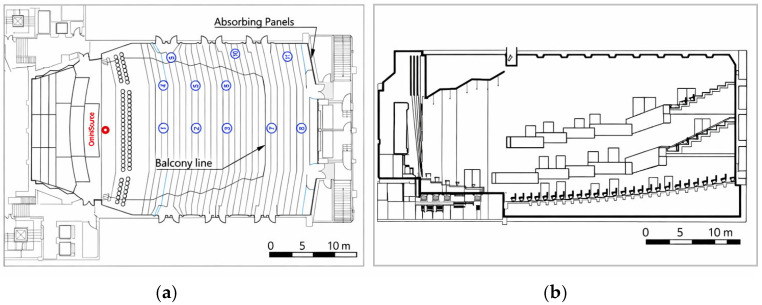
Measurement layout. (**a**) Plan view showing the omnidirectional source position (red marker) and receiver positions (blue circles, P1–P11), grouped into central, side, and under-balcony zones. The light-blue outline indicates the highlighted architectural boundary/under-balcony-related region. (**b**) Cross-sectional schematic indicating the balcony overhang geometry and under-balcony region.

Measurements were conducted in the main auditorium of the National Concert Hall (Taipei). Eleven receiver positions (P1–P11) were selected to represent (i) central open seating, (ii) side seating near the wall, and (iii) under-balcony seating, including side-under-balcony locations. Position descriptors (location/row) were used to define grouping for cross-seat comparison (Table 1). Source–receiver spatial relationships were established from full-scale architectural CAD drawings and used consistently throughout the analysis (Figure 1).

### 2.2. Measurement Procedure and Signal Chain

Measurements were conducted following ISO 3382-1 procedures for performance spaces [1], and under-balcony effects were interpreted in relation to balcony-overhang degradation mechanisms reported in prior literature [6]. An omnidirectional dodecahedron loudspeaker (Brüel & Kjær Type 4292 OmniSource, Brüel & Kjær, Nærum, Denmark ) was used as the sound source and placed on the stage at a height of 1.7 m, approximately 1 m from the stage edge (Figure 1). Excitation employed an exponential swept-sine (ESS) signal to improve signal-to-noise ratio (SNR) and suppress nonlinear distortion components; a 20 s sweep duration was adopted to ensure stable impulse-response extraction in the large-volume hall.

**Figure 2:**
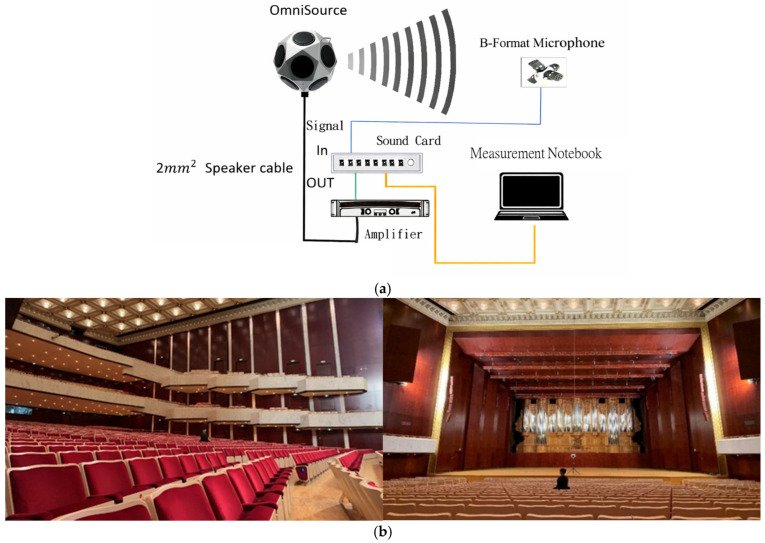
(**a**) Measurement system architecture and signal chain. Schematic diagram of the playback–capture chain used for ESS-based FOA impulse-response measurements (48 kHz/24-bit). The blue lines indicate audio signal cables, and the orange line indicates the connection cable between the sound card and the measurement notebook computer. (**b**) On-site photograph of the measurement setup. In situ photograph showing the stage-side source placement and the FOA microphone at an audience receiver position.

Spatial capture was performed using a first-order Ambisonics (FOA) scene-based microphone providing four-channel B-format signals (W, X, Y, Z), where W represents sound pressure and X/Y/Z correspond to orthogonal particle-velocity directions (left–right, front–back, vertical). Audio I/O was handled by a PreSonus Studio 192 interface expanded with a Behringer ADA8000 preamplifier, and recordings were made at 48 kHz/24-bit. At each receiver position (P1–P11), three repeated 20 s sweeps were acquired and averaged to improve SNR and reduce random variance. A schematic of the measurement system architecture and signal chain is shown in Figure 2a, and an on-site photograph of the measurement setup is provided in Figure 2b.

**Figure 3:**
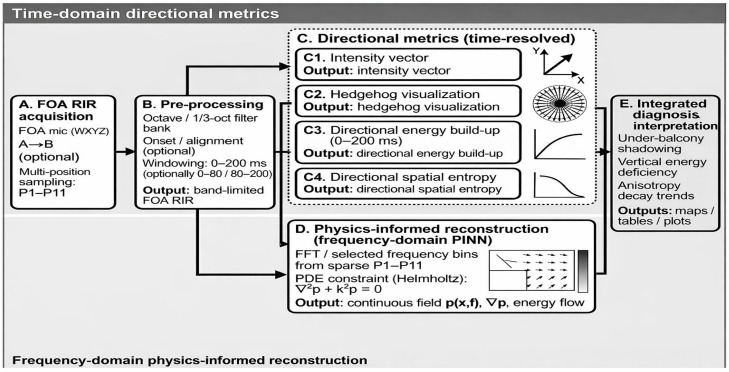
Overview of the proposed analysis pipeline from FOA RIR acquisition to directional metrics (intensity-based directional visualization, 0–200 ms directional energy build-up, and directional spatial entropy) and physics-informed reconstruction (frequency-domain PINN). The arrows indicate the intensity-based directional vectors (DoA proxies) used for reflection-direction analysis.

**Figure 4:**
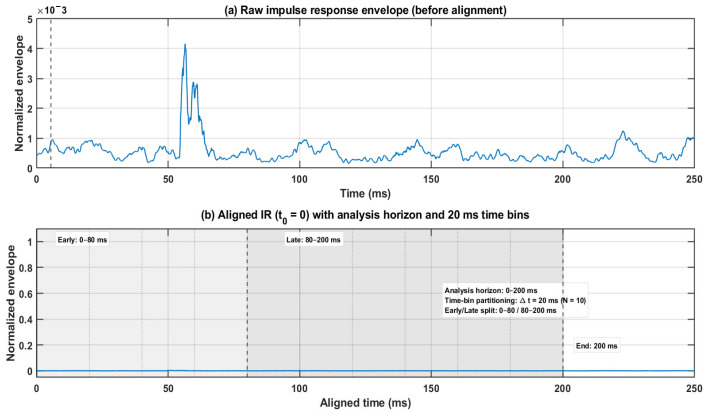
Time alignment and analysis windows used for directional feature extraction. The blue line represents the normalized impulse-response envelope: the raw envelope before alignment in panel (**a**), and the aligned envelope in panel (**b**). The figure illustrates the time-of-arrival alignment and the 0–200 ms analysis horizon with 20 ms time-bin partitioning.

The overall analysis pipeline is summarized in Figure 3, covering FOA RIR acquisition, pre-processing (time alignment and band-limiting), time-domain directional metrics (intensity-based DoA proxy, hedgehog visualization, 0–200 ms directional energy build-up, and directional spatial entropy), and the optional physics-informed reconstruction module in the frequency domain. Impulse responses were time-aligned to the direct-sound onset (time-of-arrival alignment), and all subsequent feature extraction was performed within a 0–200 ms analysis horizon. As illustrated in Figure 4, the aligned time axis was further partitioned into successive time bins, and an early/late split (0–80 ms/80–200 ms) was used for early–late discrepancy metrics.

### 2.3. FOA Representation and Intensity-Based Directional Features

Directional analysis was performed using intensity-based features derived from FOA impulse responses, consistent with parametric spatial impulse-response frameworks used in room analysis and reproduction [7,8,9,10,11,12,13]. Let acoustic pressure be approximated by the omnidirectional FOA component, p(t)∝W(t), and let the particle-velocity vector be $u(t)\propto [X(t),Y(t),Z(t){]}^{T}$, with proportionality set by the adopted FOA normalization. Instantaneous active-intensity components were computed as:

(1) $$I\left(t\right)=p\left(t\right)u\left(t\right)\propto \left[\begin{array}{c} W\left(t\right)X\left(t\right)\\ W\left(t\right)Y\left(t\right)\\ W\left(t\right)Z\left(t\right) \end{array}\right].$$

**Figure 5:**
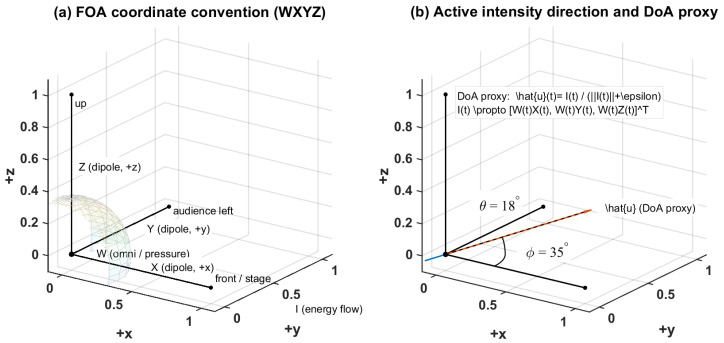
FOA coordinate convention and intensity-based DoA proxy. (**a**) FOA components (W,X,Y,Z) and axis definitions (W: omni/pressure; X,Y,Z: dipoles along +x,+y,+z). The black lines indicate the reference coordinate axes/geometric boundaries, and the red line highlights the positive axis direction used in the schematic. (**b**) Active-intensity direction and DoA proxy, where $I(t)\propto [W(t)X(t),\;W(t)Y(t),\;W(t)Z(t){]}^{T}$ and $\hat{u}\left(t\right)=\frac{I\left(t\right)}{∥I\left(t\right)∥+\epsilon }$.

The use of active and reactive intensity follows standard formulations [20,21,22]. In this study, the instantaneous active-intensity direction was used as a DoA proxy for reflection-direction analysis (Figure 5).

### 2.4. Directional Visualization Using Spherical Projection and Planar Views

For directional visualization, the DoA at each time sample was defined from the normalized active-intensity vector as

(2) $$\hat{n}\left(t\right)=\frac{I\left(t\right)}{∥I\left(t\right)∥},\mathrm{where}\;∥I\left(t\right)∥=\sqrt{I_{x}^{2}\left(t\right)+I_{y}^{2}\left(t\right)+I_{z}^{2}\left(t\right)}.$$

Here, $\hat{n}(t)$ specifies the instantaneous direction of energy flow, while ∥I(t)∥ represents its relative magnitude. Direct plotting of arrows for all time samples was avoided because dense vector rendering tends to obscure the overall directional structure, especially when early and late components are compared within the same spatial framework. Instead, the directional distribution was represented using an energy-weighted directional endpoint density, in which normalized directions were accumulated with weights proportional to ∥I(t)∥.

To improve interpretability and avoid the perspective distortion and partial occlusion associated with purely three-dimensional views, the directional distributions were primarily displayed using spherical azimuth–elevation projections. Early (0–80 ms) and late (80–200 ms) directional distributions were shown separately to allow direct comparison of the dominant arrival regions between the two temporal windows. In addition, an early–late difference map was included to summarize the redistribution of directional density between the early and late fields.

**Figure 6:**
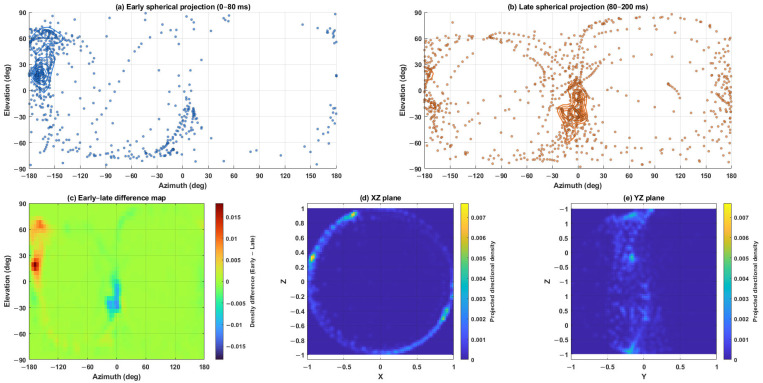
Directional distributions derived from normalized FOA active-intensity vectors. Panels (**a**,**b**) show the spherical azimuth–elevation projections of the early (0–80 ms, blue dots/curves) and late (80–200 ms, orange dots/curves) directional fields, respectively. Panel (**c**) shows the directional-density difference map (Early − Late), highlighting the redistribution of dominant arrival regions between the two temporal windows; warmer and cooler colors indicate positive and negative differences, respectively, as defined by the color bar. Panels (**d**,**e**) show the corresponding XZ and YZ planar projections, where the color scale indicates projected directional density and helps visualize the vertical structure of the directional distribution.

To further examine the vertical structure of the directional field, planar projections on the XZ and YZ planes were computed. These sectional views help assess whether the directional distribution remains concentrated near the horizontal plane or exhibits a constrained vertical spread. The combined use of spherical projection, early–late difference visualization, and vertical planar projections is intended to improve interpretability of the directional patterns while retaining physical correspondence with the FOA intensity field (Figure 6).

### 2.5. Axis-Resolved Directional Energy Build-Up (0–200 ms)

**Figure 7:**
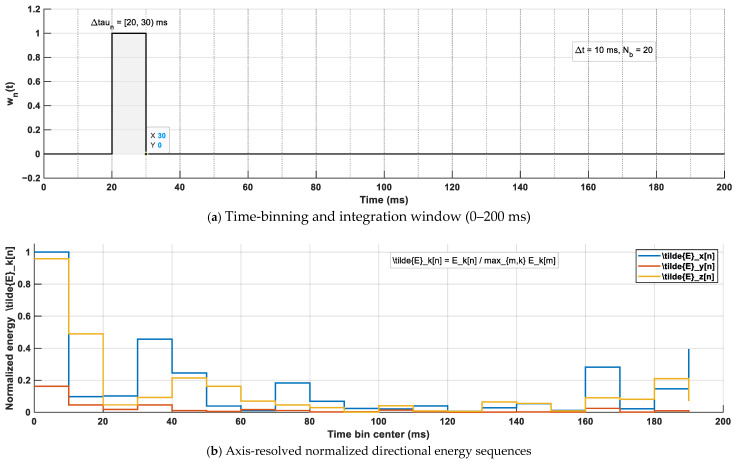
(**a**) Time-bin partitioning (0–200 ms) and rectangular window integration within Δτ_n. The gray shaded region indicates the selected time bin, and the black line represents the corresponding rectangular window function w_n(t). (**b**) Axis-resolved energy accumulation per bin and subsequent global-max normalization across axes and time bins, forming the directional energy descriptors. Explanations of the gray area and the black line have been added to the figure caption for clarity.

To quantify the temporal build-up of direction-dependent acoustic energy, the FOA intensity components $I_{x}(t)$, $I_{y}(t)$, and $I_{z}(t)$ were integrated over successive non-overlapping time bins within a 0–200 ms analysis horizon. Let the n-th time bin be defined as $\Delta \tau_{n}=[t_{n},\;t_{n+1})$, and let $w_{n}(t)$ denote a rectangular window that selects samples within $\Delta \tau_{n}$ (Figure 7a). The axis-resolved directional energy in each time bin was defined as

(3) $$E_{x}\left[n\right]=\int_{\Delta \tau_{n}}{|I_{x}\left(t\right)|}^{2}\;w_{n}\left(t\right)\;dt,E_{y}\left[n\right]=\int_{\Delta \tau_{n}}{|I_{y}\left(t\right)|}^{2}\;w_{n}\left(t\right)\;dt,E_{z}\left[n\right]=\;\int_{\Delta \tau_{n}}{|I_{z}\left(t\right)|}^{2}\;w_{n}\left(t\right)\;dt.$$

Here, ∣⋅∣ denotes the magnitude of the (potentially signed) intensity component, such that $|I_{k}(t){|}^{2}$ represents the squared magnitude used for energy accumulation on axis k∈{x,y,z}.

For cross-position comparison, the energy sequences were normalized by the global maximum across all bins and axes:

(4) $${\tilde{E}}_{k}\left[n\right]=\frac{E_{k}\left[n\right]}{\underset{m\in N,\;k\in \left\{x,y,z\right\}}{\max}E_{k}\left[m\right]},k\in \left\{x,y,z\right\},$$

where N denotes the set of time-bin indices spanning 0–200 ms. The resulting normalized sequences $\left\{{\tilde{E}}_{x}[n],\;{\tilde{E}}_{y}[n],\;{\tilde{E}}_{z}[n]\right\}$ were used as compact descriptors of the axis-resolved directional energy build-up over 0–200 ms for cross-seat comparison.

To support early–late comparisons, window-accumulated axis energies were computed by summing the bin-wise energies within the corresponding time ranges. Denoting the early window as 0–80 ms and the late window as 80–200 ms, the accumulated energies were defined as

(5) $$E_{k}^{early}=\sum_{n:\;\Delta \tau_{n}\subset [0,80\;ms)}E_{k}\left[n\right],k\in \left\{x,y,z\right\}.$$

(6) $$E_{k}^{late}=\sum_{n:\;\Delta \tau_{n}\subset [80,200\;ms)}E_{k}\left[n\right],k\in \left\{x,y,z\right\}.$$

These accumulated energies provide a consistent basis for early–late directional descriptors reported in the Results section (e.g., ratios formed from $\left\{E_{x}^{\left(.\right)},E_{y}^{\left(.\right)},E_{z}^{\left(.\right)}\right\}$).

Recommended bin settings are summarized in Table 2, and the computation procedure is illustrated in Figure 7b.

### 2.6. Directional Spatial Entropy

Directional spatial entropy was employed to quantify the degree of concentration versus spread in the angular energy distribution. For each receiver position and analysis window, the entropy was evaluated at each target frequency $f_{0}$ by constructing a direction-of-arrival (DoA) distribution from intensity-based directional samples. Specifically, the FOA components were first projected to a narrowband representation at $f_{0}$ using windowed complex demodulation. Let W(t), X(t), Y(t), and Z(t) denote the FOA components within the analysis window, and let w(t) be the selected temporal window (rectangular or Hann). The real-valued demodulated signals were obtained as

(7) $$W_{r}\left(t\right)=R\;\left\{W\left(t\right)\;w\left(t\right)\;e^{-j2\pi f_{0}t}\right\};\;X_{r}\left(t\right)=R\;\left\{X\left(t\right)\;w\left(t\right)\;e^{-j2\pi f_{0}t}\right\};\;Y_{r}\left(t\right)=R\;\left\{Y\left(t\right)\;w\left(t\right)\;e^{-j2\pi f_{0}t}\right\};\;Z_{r}\left(t\right)=R\;\left\{Z\left(t\right)\;w\left(t\right)\;e^{-j2\pi f_{0}t}\right\}.$$

Intensity components were then formed as

(8) $$I_{x}\left(t\right)=W_{r}\left(t\right)X_{r}\left(t\right),\;I_{y}\left(t\right)=W_{r}\left(t\right)Y_{r}\left(t\right),I_{z}\left(t\right)=W_{r}\left(t\right)Z_{r}\left(t\right),$$

and the instantaneous intensity magnitude proxy was defined as

(9) $$I_{mag}\left(t\right)=\sqrt{I_{x}^{2}\left(t\right)+I_{y}^{2}\left(t\right)+I_{z}^{2}\left(t\right)+\varepsilon },$$

where ε is a small constant for numerical stability. DoA unit vectors were computed from the normalized intensity direction:

(10) $$\hat{n}(t)=\frac{1}{I_{mag}\left(t\right)}\;[I_{x}(t),\;I_{y}(t),\;I_{z}(t){]}^{T}.\;$$

For entropy computation, DoA samples $\hat{n}(t)$ were assigned to a spherical partition comprising $N=N_{\phi }N_{\theta }$ angular bins (azimuth–elevation grid). A nonnegative intensity-based weight was used to accumulate contributions in each angular bin. In this study, the weight $w_{e}(t)$ was set as either $w_{e}(t)=I_{mag}(t)$ (default) or $w_{e}(t)=I_{x}^{2}(t)+I_{y}^{2}(t)+I_{z}^{2}(t)$, depending on the configuration. The accumulated bin energies were normalized to form a discrete probability mass function (PMF):

(11) $$p_{i}=\frac{\sum_{t\in B_{i}}w_{e}\left(t\right)}{\sum_{j=1}^{N}\sum_{t\in B_{j}}w_{e}\left(t\right)},i=1,\ldots ,N$$

where $B_{i}$ denotes the set of time samples whose DoA estimates fall into the i-th angular bin.

Shannon entropy was then computed from the PMF as

(12) $$H=-\sum_{i=1}^{N}p_{i}\log\left(p_{i}\right),$$

and normalized by the maximum entropy logN to obtain a bounded directional entropy index:

(13) $$H_{n}=\frac{H}{\log N}\in \left[0,1\right].$$

In this formulation, $H_{n}\to 0$ indicates strong directional concentration, that is, energy dominated by a small number of angular bins, whereas $H_{n}\to 1$ indicates a highly distributed angular energy pattern.

To characterize temporal evolution, $H_{n}$ was evaluated for both the early (0–80 ms) and late (80–200 ms) windows, denoted as $H_{n}^{early}$ and $H_{n}^{late}$, respectively. The early–late discrepancy was defined as

(14) $$\Delta H_{n}=H_{n}^{early}-H_{n}^{late}.$$

Because the normalized directional entropy depends on the adopted angular discretization, its sensitivity to bin resolution was also checked under moderate variations of $\left(N_{\phi },N_{\theta }\right)$. As expected, the absolute values of $H_{n}$ varied slightly with bin resolution. However, the main qualitative trends remained unchanged. In particular, the predominantly negative $\Delta H_{n}$ observed at the under-balcony positions was preserved across the tested binning settings, indicating that the entropy-based comparison is qualitatively robust to moderate changes in angular resolution.

**Figure 8:**
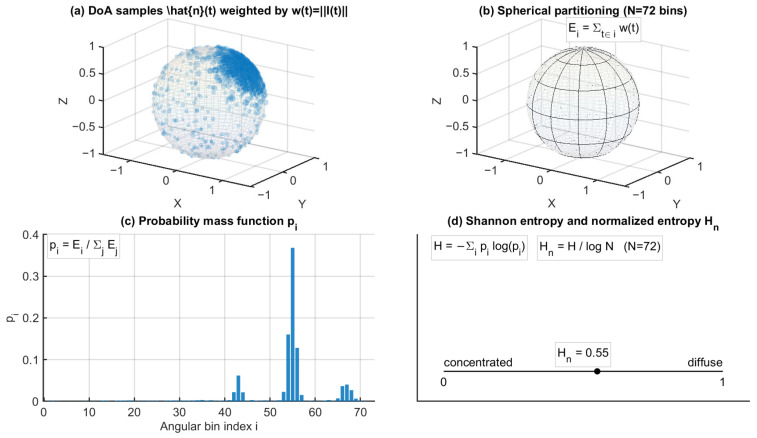
Computation workflow of directional spatial entropy from intensity-based DoA samples, including spherical angular binning, weighted probability mass function construction, and normalized entropy evaluation. In panel (**a**), the blue points represent the intensity-based DoA samples. The corresponding intensity magnitude w(t) = ||I(t)|| is used in the subsequent weighting step for constructing the probability mass function. In panel (**c**), the blue bars represent the resulting probability mass function p_i over the angular bins.

Entropy configuration details, including angular binning and weighting settings, are summarized in Table 3, and the computation workflow is illustrated in Figure 8.

### 2.7. Spatial Mapping and Physics-Regularized Reconstruction Under Sparse Measurements

#### 2.7.1. Spatial Visualization via Gaussian RBF Interpolation

To visualize spatial trends of point-wise metrics over the audience region Ω, a Gaussian radial basis function (RBF) interpolation was employed to obtain a smooth field representation from discrete receiver positions. Given a scalar metric $q(x_{m})$ evaluated at receiver locations $\{x_{m}{\}}_{m=1}^{M}$, the interpolated field $\hat{q}(x)$ was computed as

(15) $$\hat{q}\left(x\right)=\frac{\sum_{m=1}^{M}q(x_{m})exp\left(-\frac{∥x-x_{m}{∥}^{2}}{2\sigma^{2}}\right)}{\sum_{m=1}^{M}exp\left(-\frac{∥x-x_{m}{∥}^{2}}{2\sigma^{2}}\right)},$$

where σ denotes the kernel width. The interpolation was applied within the audience mask Ω to facilitate visualization of spatial variation. This RBF mapping is used solely for visualization and does not impose physical constraints.

#### 2.7.2. Frequency-Domain PINN for Physics-Regularized Sparse Sound-Field Reconstruction

To support physics-regularized interpretation under sparse measurement conditions, an optional frequency-domain physics-informed neural network (PINN) formulation was considered as a reconstruction module for the complex acoustic pressure field. For each target frequency $f_{0}$, a complex narrowband pressure proxy at the m-th receiver position $x_{m}$ was obtained from the FOA W-channel using windowed complex projection over a prescribed analysis interval (e.g., 0–200 ms):

(16) $$p^{meas}(x_{m},f_{0})=\sum_{t\in T}W(t)\;w(t)\;exp(-j2\pi f_{0}t).$$

where w(t) denotes the selected temporal window (rectangular or Hann) and T represents the discrete time samples within the analysis window. This complex quantity provides both magnitude and phase information at $f_{0}$ for the sparse receiver set.

Within the audience domain Ω, the complex pressure field $p(x,f_{0})$ was assumed to approximately satisfy the homogeneous Helmholtz equation [19,20,21]:

(17) $$\nabla^{2}p\left(x,f_{0}\right)+k^{2}p\left(x,f_{0}\right)=0,\;\;k=2\pi f_{0}/c,$$

where c is the speed of sound. A neural network $p_{\theta }(x,f_{0})$ can be used to represent the complex field via real and imaginary outputs:

(18) $$p_{\theta }\left(x,f_{0}\right)=p_{\theta }^{R}\left(x,f_{0}\right)+j\;p_{\theta }^{I}\left(x,f_{0}\right).$$

The training objective combines a data-fidelity term evaluated at the receiver positions $\{x_{m}{\}}_{m=1}^{M}$ (P1–P11 in this study) and a physics residual term evaluated at collocation points $\{x_{n}{\}}_{n=1}^{N}\subset \Omega$:

(19) $$L=\lambda_{d}\;L_{data}+\lambda_{p}\;L_{phys},$$

(20) $$L_{data}=\frac{1}{M}\sum_{m=1}^{M}{∥p_{\theta }\left(x_{m},f_{0}\right)-p^{meas}\left(x_{m},f_{0}\right)∥}_{2}^{2},$$

(21) $$L_{phys}=\frac{1}{N}\sum_{n=1}^{N}({|\nabla^{2}p_{\theta }^{R}\left(x_{n},f_{0}\right)+k^{2}p_{\theta }^{R}\left(x_{n},f_{0}\right)|}^{2}+{|\nabla^{2}p_{\theta }^{I}\left(x_{n},f_{0}\right)+k^{2}p_{\theta }^{I}\left(x_{n},f_{0}\right)|}^{2}).\;$$

The weighting parameters $\lambda_{d}$ and $\lambda_{p}$ balance measurement agreement and physics regularization.

**Figure 9:**
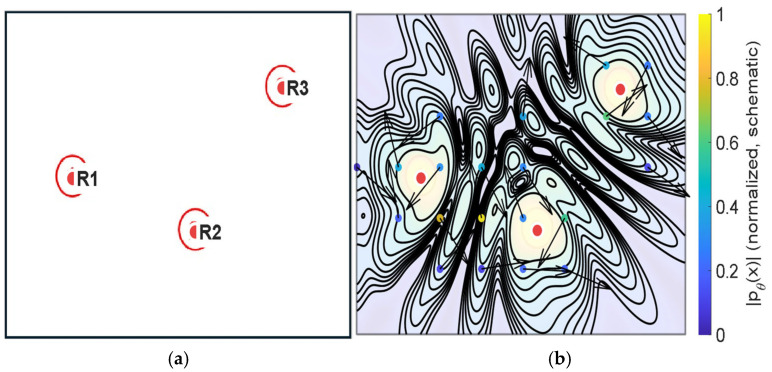
Schematic illustration of the reconstruction principle using an illustrative three-point sampling example. Panel (**a**) presents a simplified sparse measurement configuration, in which the red markers indicate the receiver locations (R1–R3). Panel (**b**) provides a conceptual wave-like continuous pressure-field visualization under physics-informed constraints. The black contour lines represent the schematic continuous field pattern, and the arrows indicate the illustrative local propagation/reconstruction direction in the conceptual field. The visualization is intended to clarify the reconstruction mechanism and does not correspond to the actual P1–P11 measurement configuration or quantitative reconstruction results.

Because explicit boundary conditions and detailed impedance models are not imposed in the present configuration, the PINN-based reconstruction should be interpreted as a physics-regularized, domain-interior approximation constrained by sparse observations, rather than a full boundary-value room simulation (e.g., Figure 9).

### 2.8. Implementation

All processing was implemented in MATLAB R2023a (Academic Use, The MathWorks, Natick, MA, USA),including impulse-response extraction, FOA directional metrics, entropy computation, and PINN training/inference.

## 3. Results

**Figure 10:**
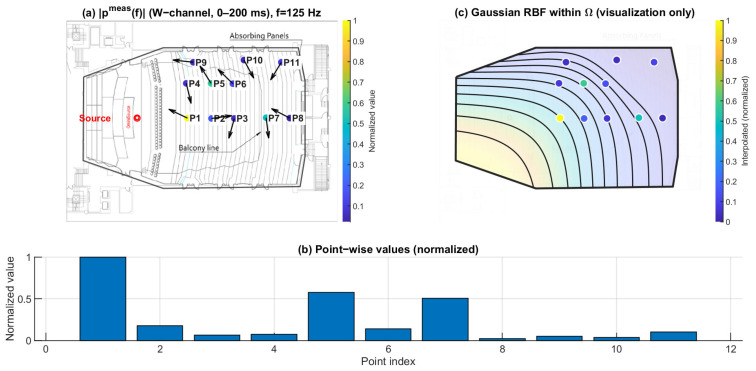
Spatial distribution of $|p^{meas}(f)|$ at 125 Hz. (**a**) Point-wise normalized $|p^{meas}(f)|$ (FOA W-channel, 0–200 ms) mapped to the plan view; the arrows indicate the intensity-based directional vectors at the receiver positions. (**b**) Normalized values across P1–P11. (**c**) Gaussian RBF interpolation within Ω for visualization of spatial trends only; the black lines represent schematic RBF contour lines, and the colored dots indicate the corresponding point-wise normalized values projected onto the audience domain, with colors referenced to the color bar.

**Figure 11:**
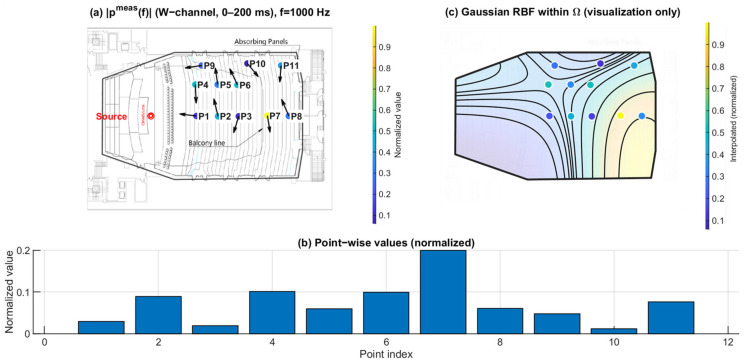
Spatial distribution of $|p^{meas}(f)|$ at 1 kHz. (**a**) Point-wise normalized $|p^{meas}(f)|$ (FOA W-channel, 0–200 ms) mapped to the plan view; the arrows indicate the intensity-based directional vectors at the receiver positions. (**b**) Normalized values across P1–P11. (**c**) Gaussian RBF interpolation within Ω for visualization of spatial trends only; the black lines represent schematic RBF contour lines, and the colored dots indicate the corresponding point-wise normalized values projected onto the audience domain, with colors referenced to the color bar.

**Figure 12:**
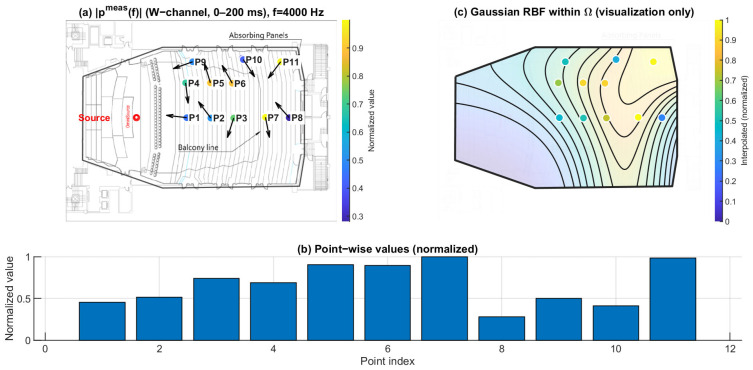
Spatial distribution of $|p^{meas}(f)|$ at 4 kHz. (**a**) Point-wise normalized $|p^{meas}(f)|$ (FOA W-channel, 0–200 ms) mapped to the plan view; the arrows indicate the intensity-based directional vectors at the receiver positions. (**b**) Normalized values across P1–P11. (**c**) Gaussian RBF interpolation within Ω for visualization of spatial trends only; the black lines represent schematic RBF contour lines, and the colored dots indicate the corresponding point-wise normalized values projected onto the audience domain, with colors referenced to the color bar.

**Figure 13:**
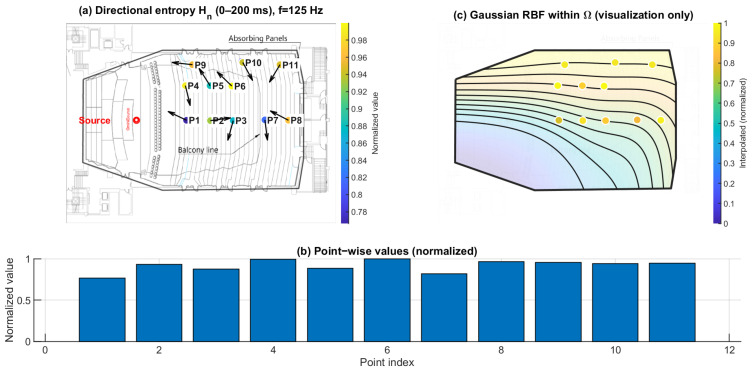
Spatial distribution of directional entropy $H_{n}$ at 125 Hz. (**a**) Point-wise normalized $H_{n}$ (0–200 ms) mapped to the plan view; the arrows indicate the intensity-based directional vectors at the receiver positions. (**b**) Normalized values across P1–P11. (**c**) Gaussian RBF interpolation within Ω for visualization of spatial trends only; the black lines represent schematic RBF contour lines, and the colored dots indicate the corresponding point-wise normalized values projected onto the audience domain, with colors referenced to the color bar.

**Figure 14:**
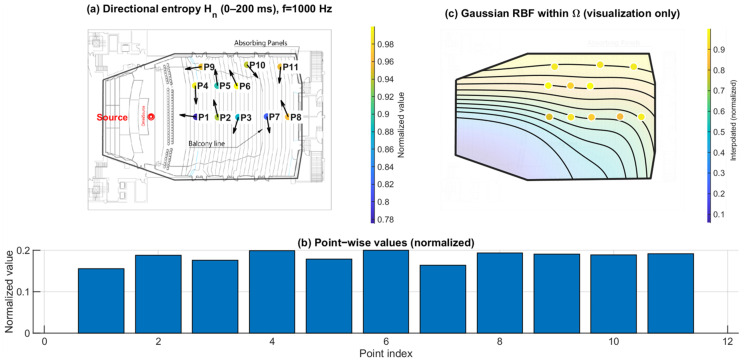
Spatial distribution of directional entropy $H_{n}$ at 1 kHz. (**a**) Point-wise normalized $H_{n}$ (0–200 ms) mapped to the plan view; the arrows indicate the intensity-based directional vectors at the receiver positions. (**b**) Normalized values across P1–P11. (**c**) Gaussian RBF interpolation within Ω for visualization of spatial trends only; the black lines represent schematic RBF contour lines, and the colored dots indicate the corresponding point-wise normalized values projected onto the audience domain, with colors referenced to the color bar.

**Figure 15:**
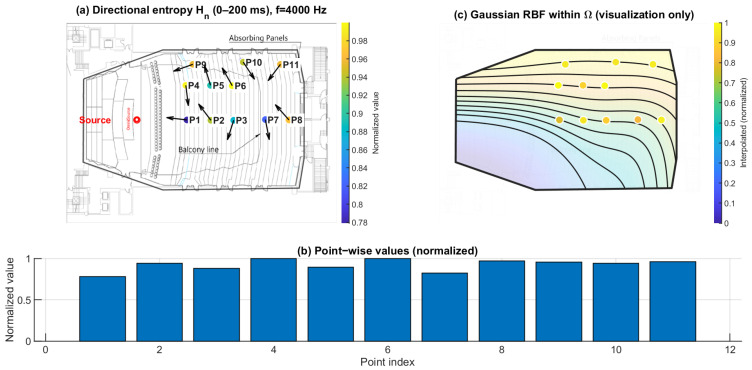
Spatial distribution of directional entropy $H_{n}$ at 4 kHz. (**a**) Point-wise normalized $H_{n}$ (0–200 ms) mapped to the plan view; the arrows indicate the intensity-based directional vectors at the receiver positions. (**b**) Normalized values across P1–P11. (**c**) Gaussian RBF interpolation within Ω for visualization of spatial trends only; the black lines represent schematic RBF contour lines, and the colored dots indicate the corresponding point-wise normalized values projected onto the audience domain, with colors referenced to the color bar.

**Figure 16:**
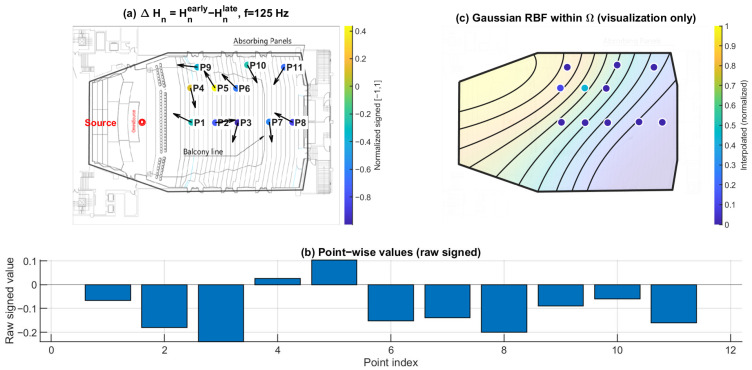
Early–late directional entropy discrepancy $\Delta H_{n}$ at 125 Hz. (**a**) Point-wise $\Delta H_{n}=H_{n}^{early}-H_{n}^{late}$ (early: 0–80 ms; late: 80–200 ms) mapped to the plan view (normalized signed values); the arrows indicate the intensity-based directional vectors at the receiver positions. (**b**) Raw signed $\Delta H_{n}$ values across P1–P11. (**c**) Gaussian RBF interpolation within Ω for visualization of spatial trends only; the black lines represent schematic RBF contour lines, and the colored dots indicate the corresponding point-wise values projected onto the audience domain, with colors referenced to the color bar.

**Figure 17:**
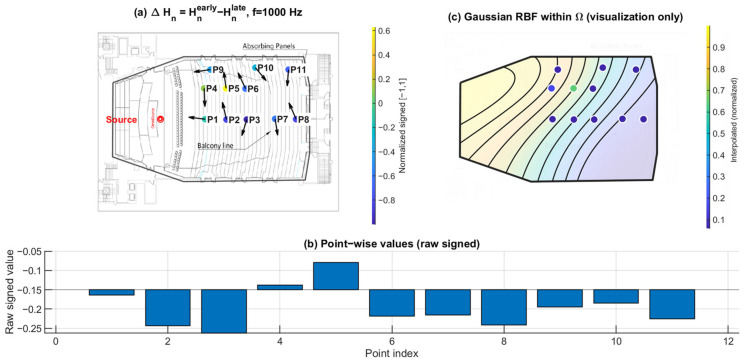
Early–late directional entropy discrepancy $\Delta H_{n}$ at 1 kHz. (**a**) Point-wise $\Delta H_{n}=H_{n}^{early}-H_{n}^{late}$ (early: 0–80 ms; late: 80–200 ms) mapped to the plan view (normalized signed values); the arrows indicate the intensity-based directional vectors at the receiver positions. (**b**) Raw signed $\Delta H_{n}$ values across P1–P11. (**c**) Gaussian RBF interpolation within Ω for visualization of spatial trends only; the black lines represent schematic RBF contour lines, and the colored dots indicate the corresponding point-wise values projected onto the audience domain, with colors referenced to the color bar.

**Figure 18:**
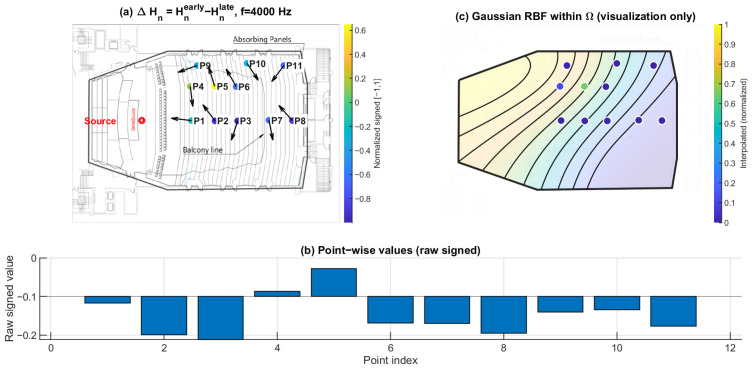
Early–late directional entropy discrepancy $\Delta H_{n}$ at 4 kHz. (**a**) Point-wise $\Delta H_{n}=H_{n}^{early}-H_{n}^{late}$ (early: 0–80 ms; late: 80–200 ms) mapped to the plan view (normalized signed values); the arrows indicate the intensity-based directional vectors at the receiver positions. (**b**) Raw signed $\Delta H_{n}$ values across P1–P11. (**c**) Gaussian RBF interpolation within Ω for visualization of spatial trends only; the black lines represent schematic RBF contour lines, and the colored dots indicate the corresponding point-wise values projected onto the audience domain, with colors referenced to the color bar.

**Figure 19:**
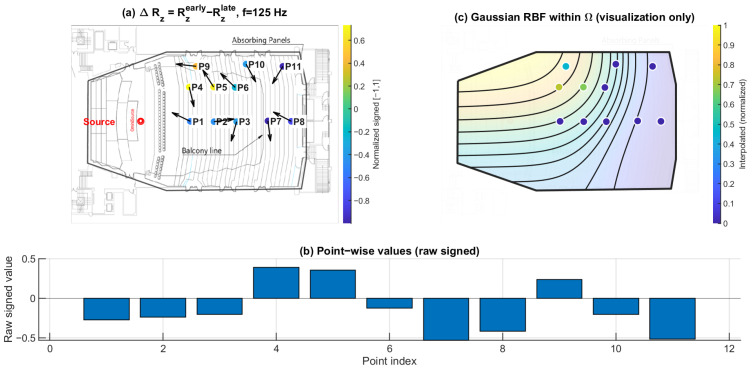
Early–late vertical energy discrepancy $\Delta R_{z}$ at 125 Hz. (**a**) Point-wise $\Delta R_{z}=R_{z}^{early}-R_{z}^{late}$ (early: 0–80 ms; late: 80–200 ms) mapped to the plan view (normalized signed values); the arrows indicate the intensity-based directional vectors at the receiver positions. (**b**) Raw signed $\Delta R_{z}$ values across P1–P11. (**c**) Gaussian RBF interpolation within Ω for visualization of spatial trends only; the black lines represent the schematic contour lines of the interpolated field, and the colored dots indicate the corresponding point-wise values projected onto the audience domain, with colors referenced to the color bar.

**Figure 20:**
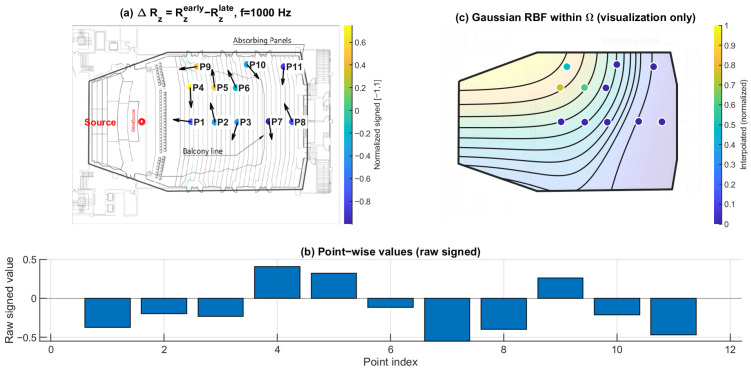
Early–late vertical energy discrepancy $\Delta R_{z}$ at 1 kHz. (**a**) Point-wise $\Delta R_{z}=R_{z}^{early}-R_{z}^{late}$ (early: 0–80 ms; late: 80–200 ms) mapped to the plan view (normalized signed values); the arrows indicate the intensity-based directional vectors at the receiver positions. (**b**) Raw signed $\Delta R_{z}$ values across P1–P11. (**c**) Gaussian RBF interpolation within Ω for visualization of spatial trends only; the black lines represent the schematic contour lines of the interpolated field, and the colored dots indicate the corresponding point-wise values projected onto the audience domain, with colors referenced to the color bar.

**Figure 21:**
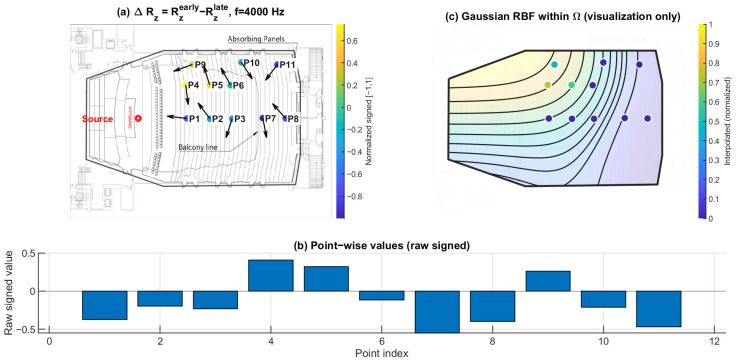
Early–late vertical energy discrepancy $\Delta R_{z}$ at 4 kHz. (**a**) Point-wise $\Delta R_{z}=R_{z}^{early}-R_{z}^{late}$ (early: 0–80 ms; late: 80–200 ms) mapped to the plan view (normalized signed values); the arrows indicate the intensity-based directional vectors at the receiver positions. (**b**) Raw signed $\Delta R_{z}$ values across P1–P11. (**c**) Gaussian RBF interpolation within Ω for visualization of spatial trends only; the black lines represent the schematic contour lines of the interpolated field, and the colored dots indicate the corresponding point-wise values projected onto the audience domain, with colors referenced to the color bar.

Results are reported at three representative frequencies (125 Hz, 1 kHz, and 4 kHz) using the directional metrics defined in Section 2. The FOA coordinate convention and intensity-based DoA proxy follow Figure 5, the early/late windowing and axis-resolved energy accumulation follow Figure 4 and Figure 7, and the directional entropy $H_{n}$ follows the binning and normalization procedure in Figure 8 (Table 3). Figure 6 provides a qualitative illustration of how the directional energy distribution differs between the early and late windows and how the corresponding vertical structure can be assessed using the XZ and YZ projections. For each frequency and metric, the corresponding figure contains: (a) point-wise values mapped to the plan view, (b) normalized point-wise values across P1–P11, and (c) Gaussian RBF interpolation within the audience domain Ω for visualization of spatial trends only (Section 2.7.1). Unless otherwise stated, quantitative cross-seat comparisons are primarily drawn from panel (b) of Figure 10, Figure 11, Figure 12, Figure 13, Figure 14, Figure 15, Figure 16, Figure 17, Figure 18, Figure 19, Figure 20 and Figure 21, while panel (c) is used only to illustrate spatial gradients.

### 3.1. Measured Pressure Proxy $|p^{meas}(f)|$ (W-Channel, 0–200 ms)

Figure 10, Figure 11 and Figure 12 show the spatial distribution of the measured pressure-proxy magnitude $|p^{meas}(f)|$ (W-channel, 0–200 ms) at 125 Hz, 1 kHz, and 4 kHz. The under-balcony receivers correspond to P7–P11 (Table 1). In each figure, panel (b) provides the point-wise comparison across P1–P11, while panel (c) is a Gaussian RBF visualization within Ω.

At 125 Hz (Figure 10), $|p^{meas}(f)|$ varies across seats and the under-balcony region tends to show lower magnitudes than open seating. At 1 kHz (Figure 11), the distribution becomes more seat-dependent, and the under-balcony region contains both relatively high- and low-magnitude points. At 4 kHz (Figure 12), the contrast between open and under-balcony seats becomes clearer again, while variability within P7–P11 remains evident. These results indicate that under-balcony effects cannot be characterized by a uniform level reduction. Instead, the response depends strongly on receiver location, especially in the mid and high bands.

### 3.2. Directional Spatial Entropy $H_{n}$ (0–200 ms): Angular Spread of Energy

Figure 13, Figure 14 and Figure 15 report the normalized directional spatial entropy $H_{n}$ over 0–200 ms at 125 Hz, 1 kHz, and 4 kHz. Across all three frequencies, $H_{n}$ is generally high, indicating that the angular energy distribution accumulated within 0–200 ms is broadly spread at most seats. However, consistent seat-to-seat differences remain, with several positions showing lower $H_{n}$, i.e., a more concentrated directional distribution.

At 125 Hz (Figure 13), the entropy field already exhibits spatial structure. At 1 kHz (Figure 14), the seat dependence becomes more distinct. At 4 kHz (Figure 15), the patterns highlight stronger directional constraints in geometrically shadowed locations. Compared with $|p^{meas}(f)|$, $H_{n}$ provides complementary information by describing how energy is distributed across arrival directions rather than only indicating magnitude.

### 3.3. Early–Late Entropy Discrepancy $\Delta H_{n}$: Temporal Evolution of Directional Diffuseness

Figure 16, Figure 17 and Figure 18 show the early–late entropy discrepancy $\Delta H_{n}=H_{n}^{early}-H_{n}^{late}$, using early 0–80 ms and late 80–200 ms windows, at 125 Hz, 1 kHz, and 4 kHz. Across the receiver set, $\Delta H_{n}$ is predominantly negative at all three frequencies. This indicates that the early field is more directionally concentrated than the late field, consistent with early arrivals being governed by fewer dominant reflection directions.

A systematic under-balcony tendency is observed: P7–P11 exhibit more negative $\Delta H_{n}$ than open seating at each of the three frequencies. This implies that the early directional structure beneath the balcony is more constrained relative to the late field, consistent with the overhang suppressing or weakening specific early reflection paths.

### 3.4. Early–Late Vertical Energy Discrepancy $\Delta R_{z}$: Selective Loss of Early Vertical Contribution Under the Balcony

Figure 19, Figure 20 and Figure 21 present the early–late discrepancy of the vertical energy ratio, $\Delta R_{z}=R_{z}^{early}-R_{z}^{late}$, at 125 Hz, 1 kHz, and 4 kHz. This metric isolates changes in the vertical contribution between early and late windows and therefore targets direction-specific imbalance rather than overall magnitude variation.

Open seating (P1–P6) shows $\Delta R_{z}$ values clustered near zero across the three frequencies. In contrast, the under-balcony group (P7–P11) shows consistently negative $\Delta R_{z}$, with the most negative values repeatedly occurring within the under-balcony set. This indicates a selective reduction in early vertical contribution beneath the balcony, consistent with weakened ceiling- and overhead-related early reflections, while later energy (80–200 ms) becomes more redistributed.

## 4. Discussion

### 4.1. Direction-Specific Mechanism of Under-Balcony Degradation

The present results indicate that under-balcony behavior cannot be adequately explained by a uniform attenuation mechanism. Pressure-proxy maps $|p^{meas}(f)|$ (Figure 10, Figure 11 and Figure 12) exhibit substantial seat-to-seat variability within P7–P11, indicating localized reinforcement and shadowing rather than a spatially homogeneous level loss. In contrast, the early–late vertical discrepancy $\Delta R_{z}$ (Figure 19, Figure 20 and Figure 21) provides a stable under-balcony signature across 125 Hz, 1 kHz, and 4 kHz: P7–P11 consistently show negative $\Delta R_{z}$, whereas open seating (P1–P6) clusters near zero. This pattern supports a direction-selective reduction in early vertical contribution (0–80 ms) relative to the late field (80–200 ms) in the shadowed zone, consistent with an overhang mechanism that weakens ceiling- and overhead-related early reflections.

### 4.2. Directional Entropy and Early–Late Evolution of Anisotropy

Directional spatial entropy $H_{n}$ (Figure 13, Figure 14 and Figure 15) remains generally high over 0–200 ms, indicating that the accumulated angular-energy distribution is broadly spread for most seats. However, the early–late discrepancy $\Delta H_{n}$ (Figure 16, Figure 17 and Figure 18) is predominantly negative and becomes more negative under the balcony. This implies that the early field is more directionally concentrated than the late field, and that this early directional constraint is stronger in P7–P11. Such behavior is consistent with early arrivals being governed by a limited set of viable reflection paths, while later energy becomes progressively redistributed by higher-order reflections.

The qualitative directional views in Figure 6 are consistent with this interpretation. The early and late spherical projections show that the dominant directional regions differ between the two temporal windows, indicating that the directional energy distribution is redistributed rather than preserved as a stable angular pattern. This observation supports the interpretation of negative $\Delta H_{n}$, namely, that the early field is constrained by fewer dominant arrival directions, whereas the late field becomes more directionally spread. In particular, the YZ projection indicates a relatively constrained vertical spread, supporting the view that the under-balcony sound field is affected not only by seat-dependent magnitude variation but also by restricted vertical directional structure. The XZ projection further suggests that much of the directional energy remains concentrated near the horizontal plane rather than being broadly distributed over elevation.

Together with $\Delta R_{z}$, $\Delta H_{n}$ separates temporal redistribution effects from direction-specific deficits. While $\Delta H_{n}$ reflects how directional diffuseness evolves from the early to the late field, $\Delta R_{z}$ directly captures whether vertical energy contribution is selectively weakened in the early response. Considered jointly, these metrics indicate that the under-balcony condition is characterized by both stronger early anisotropy and limited vertical-energy development, rather than by a simple uniform attenuation of sound level.

### 4.3. Frequency Dependence Across 125 Hz, 1 kHz, and 4 kHz

Across the three representative frequencies, the observed patterns are consistent with a transition from diffraction-influenced behavior at low frequency to shadowing-dominated behavior at mid and high frequencies. At 125 Hz, spatial differences are present but are partially mitigated by long wavelengths. At 1 kHz and 4 kHz, the under-balcony region exhibits clearer directional constraints and stronger early vertical-energy loss, consistent with reduced access to specular overhead reflections and increased sensitivity to geometric occlusion. The persistence of negative $\Delta R_{z}$ across frequencies further suggests that the under-balcony deficit is not a narrowband artifact but a robust directional feature of the early response.

### 4.4. Diagnostic Implications Beyond Scalar Metrics

Scalar descriptors can indicate seat-dependent variation but cannot directly reveal which directional components are selectively affected. The present results show that $|p^{meas}(f)|$ alone is insufficient to distinguish between uniform attenuation and direction-specific imbalance, particularly when within-zone variability is large. By contrast, the proposed set of direction-resolved metrics provides complementary diagnostic information: $H_{n}$ characterizes angular spread over 0–200 ms, $\Delta H_{n}$ captures early-to-late redistribution, and $\Delta R_{z}$ directly exposes selective early vertical-energy deficiency. This separation improves interpretability and supports mechanism-driven diagnosis of under-balcony acoustic issues.

### 4.5. Limitations

This study is based on a sparse receiver set (11 positions). Spatial maps in panels (c) of Figure 10, Figure 11, Figure 12, Figure 13, Figure 14, Figure 15, Figure 16, Figure 17, Figure 18, Figure 19, Figure 20 and Figure 21 are generated by Gaussian RBF interpolation for visualization only; therefore, quantitative interpretation should rely primarily on the point-wise comparisons (panel (b)). The DoA proxy is intensity-based and evaluated per frequency using a narrowband representation; results can be affected by the choice of early/late windows and the temporal weighting window. Finally, the present evaluation focuses on three representative frequencies; extending the analysis to additional bands would further clarify frequency-dependent transitions.

In addition, the normalized directional entropy $H_{n}$ depends to some extent on the adopted angular binning $\left(N_{\phi },N_{\theta }\right)$. Although absolute values vary slightly with bin resolution, the principal cross-seat trends reported here—most notably the consistently negative $\Delta H_{n}$ in the under-balcony group—remained stable under moderate changes in binning density.

## 5. Conclusions

This study presented a FOA-based directional analysis framework for diagnosing spatial acoustic degradation in under-balcony seating areas. By combining intensity-based direction-of-arrival estimation, axis-resolved directional energy build-up, and directional spatial entropy analysis, the proposed approach enables mechanism-oriented interpretation of directional reflection structures that are not captured by conventional scalar metrics.

The experimental results lead to several key findings. First, the pressure-proxy magnitude $|p^{meas}(f)|$ exhibits strong seat-to-seat variability in the under-balcony region, indicating that the observed degradation cannot be explained by a spatially uniform attenuation mechanism. Second, directional spatial entropy analysis shows that the angular energy distribution remains broadly spread over the full 0–200 ms interval but exhibits localized reductions that suggest directional constraints in specific seating locations. Third, the early–late entropy discrepancy $\Delta H_{n}$ is predominantly negative across all analyzed frequencies, indicating stronger directional concentration in the early sound field. Finally, the early–late vertical energy discrepancy $\Delta R_{z}$ provides the most consistent under-balcony signature, with negative values observed at all shadowed positions, revealing a systematic reduction in early vertical reflection contributions.

The proposed framework therefore provides a physically interpretable method for identifying direction-specific early-reflection deficits in geometrically constrained listening zones. Beyond under-balcony scenarios, the same methodology can be applied to other architectural configurations where reflection paths may be partially blocked, such as deep overhangs, alcoves, or recessed seating areas.

Future work will extend the analysis to denser spatial sampling and broader frequency coverage to better characterize frequency-dependent transitions in directional behavior. In addition, intervention studies will investigate how architectural or electroacoustic modifications influence $\Delta R_{z}$ and $\Delta H_{n}$, enabling the proposed metrics to serve as design-feedback indicators for targeted acoustic improvements.

## Figures and Tables

**Table 1:** Receiver positions and zone label.

Microphone Position	Location Description
P1	Central area, 7th row, center of audience seating
P2	Central area, 12th row, center of audience seating
P3	Central area, 17th row, center of audience seating
P4	Side area, 7th row, near wall
P5	Side area, 12th row, near wall
P6	Side area, 17th row, near wall
P7	Under the balcony, 23rd row, center
P8	Under the balcony, 30th row, center
P9	Side area under balcony, 9th row, near wall
P10	Side area under balcony, 20th row, near wall
P11	Side area under balcony, 27th row, near wall

**Table 2:** Time-bin configuration for axis-resolved directional energy build-up (0–200 ms).

Parameter	Value
Analysis horizon	0–200 ms
Bin width Δt	(e.g., 5 ms/10 ms)
Number of bins	$N_{b}=\frac{200}{\Delta t}$
Window type	Rectangular $w_{n}\left(t\right)$
Normalization	Global max across axes and bins

**Table 3:** Configuration of directional spatial entropy $H_{n}$.

Item	Setting
Spherical partition	Azimuth–elevation grid (uniform binning): azimuth ϕ∈[−π,π] divided into $N_{\phi }$ bins; elevation θ∈[−π/2,π/2] divided into $N_{\theta }$ bins.
Number of bins	$N=N_{\phi }N_{\theta }$; in this study $N_{\phi }=24,\;$$N_{\theta }=12,$ hence N=288.
Weighting function	Intensity-based nonnegative weight $w_{e}(t)$: default $w_{e}(t)=I_{mag}(t)=\sqrt{I_{x}^{2}(t)+I_{y}^{2}(t)+I_{z}^{2}(t)+\varepsilon };$ alternatively $w_{e}(t)=I_{x}^{2}(t)+I_{y}^{2}(t)+I_{z}^{2}(t).$
Time window	$H_{n}$ evaluated over 0–200 ms (full window) and separately over early 0–80 ms and late 80–200 ms windows for $\Delta H_{n}.$ Temporal weighting uses w(t) = Hann (default) or rectangular.
Frequency handling	Per-frequency narrowband evaluation at target frequency $f_{0}$ using windowed complex demodulation (single-frequency projection): FOA components are demodulated by $e^{-j2\pi f_{0}t}$ and the real parts are used to form intensity components before binning.
Normalization	$H_{n}=H/logN,$ with $H=-\sum_{i=1}^{N}p_{i}log(p_{i}),$ yielding $H_{n}\in [0,1].$

## Data Availability

The data presented in this study are available from the corresponding author upon reasonable request.
